# Anti-hepatitis C virus threshold value in predicting hepatitis C virus viremia in hemodialysis patients

**DOI:** 10.1590/1806-9282.20250184

**Published:** 2025-09-19

**Authors:** Vahibe Aydın Sarıkaya, Uğur Ayan, Sebahat Aksaray, Selami Erdinç, Burak Sarıkaya, Recep Balık, Seniha Şenbayrak, Serpil Erol, Asuman İnan, Nurgül Ceran

**Affiliations:** 1 Haydarpaşa Numune Training and Research Hospital, Department of Infectious Diseases and Clinical Microbiology – Istanbul, Turkey.; 2 Göztepe Prof. Dr. Süleyman Yalçın City Hospital, Department of Medical Microbiology, ISLAB-2 Regional Laboratory – Istanbul, Turkey.; 3 Haydarpaşa Numune Training and Research Hospital, Department of Medical Microbiology – Istanbul, Turkey.; 4 University of Health Sciences, Sultan 2. Abdulhamid Han Training and Research Hospital, Department of Infectious Diseases and Clinical Microbiology – Istanbul, Turkey.

**Keywords:** Hemodialysis, Hepatitis C virus, Viremia, Genotype

## Abstract

**OBJECTIVE:**

Hepatitis C virus prevalence is higher in hemodialysis patients than in the general population. It is recommended that patients who are detected to be anti-hepatitis C virus seropositive should be dialyzed in separate machines. In patients requiring urgent hemodialysis treatment, anti-hepatitis C virus seropositivity may cause confusion and delay in dialysis sessions.

**METHODS:**

The aim of the study was to determine the most appropriate signal-to-cutoff value to predict hepatitis C virus viremia in hemodialysis patients and to evaluate the effect of genotype differences. A total of 12,280 anti-hepatitis C virus results from hemodialysis patients between 2021 and 2024 were examined.

**RESULTS:**

The mean age of 563 patients included in the study was 57 years, and 330 (58.6%) were male. Of the 563 patients, 68 (12.07%) were true hepatitis C virus patients. The mean age of hepatitis C virus-ribonucleic acid(+) patients was higher than that of the hepatitis C virus-ribonucleic acid(-) group (p<0.018). Anti-hepatitis C virus signal-to-cutoff value was >1 in all true hepatitis C virus patients. Hepatitis C virus-ribonucleic acid was accepted as the gold standard to determine the best threshold value in receiver operating characteristic curve analysis, and the most appropriate signal-to-cutoff value was found to be 2.23. Sensitivity was 98.5%, specificity was 87.1%, positive predictive value was 51.2%, and negative predictive value was 99.8%. 49 (85.96%) of the patients were identified as genotype(-1; the most common subtype was genotype-1b (n=43).

**CONCLUSION:**

Anti-hepatitis C virus negativity is a reliable result in hemodialysis patients. If anti-hepatitis C virus signal-to-cutoff ≥2.23 is detected, confirmation by direct hepatitis C virus-ribonucleic acid testing is recommended. In hemodialysis patients with anti-hepatitis C virus signal-to-cutoff values between 1 and 2.23, false positivity should be considered first, and confirmatory tests should be performed if anti-hepatitis C virus is reactive in a second sample.

## INTRODUCTION

Hepatitis C virus (HCV) is a virus that can cause chronic hepatitis^1^. HCV infection is more common in hemodialysis (HD) patients than in the general population. In some countries, the prevalence ranges from 5 to 60%^1^. In hemodialysis patients, HCV infection screening is recommended before the first dialysis session and periodically every 6 months^2^. Dialysis of anti-HCV reactive patients on separate dialysis machines is mandatory in Turkey and many other countries^3^.

In the HCV diagnostic algorithm, it is recommended to check anti-HCV antibody as a screening test, and if the result is reactive, HCV RNA testing is recommended for definitive diagnosis^4,5^. In anti-HCV tests, the signal-to-cutoff (S/Co) value, which is the ratio of the optical density of the sample to the cut-off value, is used as the reactivity threshold. A value of S/Co ≥1 is considered positive according to the manufacturer's recommendation^6^. In countries with low HCV prevalence, the anti-HCV test can often give false positivity with a result slightly above the cut-off value^7^. It is also known that the anti-HCV test result is more highly reactive in HD patients than in the general population^8^.

Anti-HCV positivity in patients requiring emergency hemodialysis treatment may cause confusion and delay of the dialysis sessions. In addition, a false positive test result leads to unnecessary repeat testing for laboratories, increased costs due to the need for confirmatory testing, and psychological stress for patients^6^.

In this retrospective study, it was planned to determine the most appropriate anti-HCV S/Co value for predicting HCV viremia in patients receiving hemodialysis treatment and to evaluate the effect of HCV genotype differences on this situation.

## METHODS

This study was conducted with the approval of the Ethics Committee for Clinical Researches at Haydarpasa Numune Training and Research Hospital Clinical Research Ethics Committee (16.07.2024/96).

The results of patients who received hemodialysis treatment between January 2021 and January 2024 and who underwent anti-HCV, HCV RNA, and HCV genotyping were evaluated retrospectively.

Patients younger than 18 years of age, not receiving hemodialysis treatment, and not having HCV RNA studied simultaneously with anti-HCV were excluded.

During the 3-year study period, a total of 12,280 HCV tests were performed in hemodialysis patients. After excluding repeat tests and patients under 18 years of age, demographic data were calculated for 563 HD patients whose complete data were available. Age, gender, anti-HCV S/Co value, HCV RNA level, and HCV genotype results of these patients were entered into Excel, and statistical analysis was performed.

Anti-HCV tests were performed with the "Electrochemiluminescence Immunoassay" method in accordance with the manufacturer's recommendations, using the third-generation "Elecsys Anti-HCV II" kit (Roche Diagnostics, Germany) on the Roche cobas^®^ e 801 device.

Viral nucleic acid isolation for HCV-RNA detection using the "QIAsymphony SP/AS" system and "QIAsymphony DSP virus/pathogen midi kit" (Qiagen, Germany); polymerase chain reaction (PCR) was performed using the "Artus HCV QS-RGQ" kit (Qiagen, Germany) with the "Rotor-Gene Q" system in accordance with the manufacturer's instructions.

HCV genotyping was determined qualitatively with the "Sacace HCV Genotype Plus Real-TM" kit (Sacace biotechnologies, Italy) using the "Rotor-Gene Q" system and real-time PCR method.

### Statistical analysis

Data were evaluated in the IBM Statistical Package for Social Sciences Statistics 25.0 statistical package program. Descriptive statistics were given as number of units (n), percentage (%), mean±standard deviation, and median (Q1–Q3) values. Pearson chi-square and Fisher's exact test were used in the evaluation of categorical variables. The normal distribution of continuous variables was evaluated by Shapiro-Wilk, normality test, and Q-Q graphs. The Mann-Whitney U test was used to compare the continuous variables of two groups, and the Kruskal-Wallis analysis was used to compare the continuous variables of three or more groups. The Dunn-Bonferroni test was used as a multiple comparison test. The performance of anti-HCV in identifying true HCV patients was evaluated by drawing ROC (receiver operating characteristic) curves. Threshold values were determined using the Youden index. Sensitivity, specificity, positive predictive value, and negative predictive value were calculated based on the threshold values. A value of p<0.05 was considered statistically significant.

## RESULTS

The median age of the 563 patients included in the study was 57 years, with 330 (58.6%) being male (Table 1).

**Table 1:** Comparison of demographic and clinical characteristics of cases according to hepatitis C virus RNA.

Variables		HCV-RNA n (%)			p-value
		Total (n: 563)	HCV-RNA (+) 68 (12.1%)	HCV-RNA (-) 495 (87.9%)	
Age (year), mean±SD		56.8±14.98	61.26±13.37	56.14±15.1	**0.018**
Gender	Female	233 (41.4%)	31 (13.3%)	202 (86.7%)	0.453
	Male	330 (58.6%)	37 (11.2%)	293 (88.8%)	
Anti-HCV, S/Co ratio Median (Q1–Q3)		0.045 (0.039–0.199)	49.9 (32.2–83.4)	0.043 (0.038–0.054)	**<0.0001**

n: number of cases; SD: standard deviation; anti-HCV: hepatitis C virus antibody; HCV-RNA: hepatitis C virus ribonucleic acid; Q1–Q3: interquartile range; S/Co: signal-to-cutoff. The bold values indicate statistically significant p-values.

**Figure 1:**
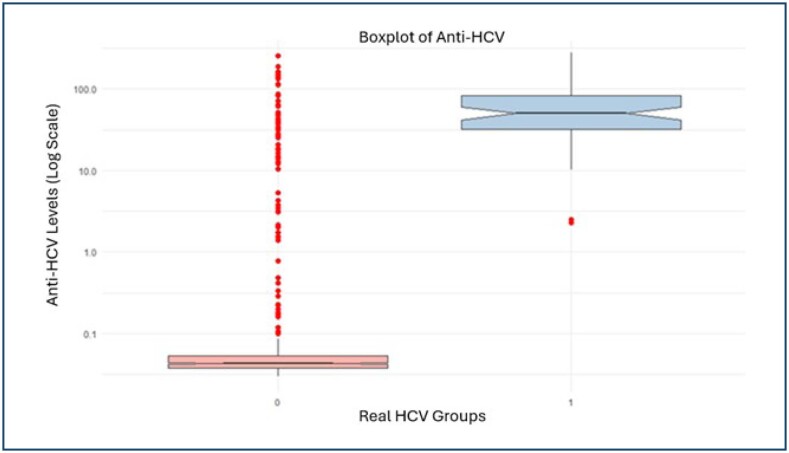
Boxplot analysis of anti-hepatitis C virus signal-to-cutoff values according to real hepatitis C virus status.

Of 563 patients, 68 (12.07%) were true HCV patients. Anti-HCV S/Co value >1 in all true HCV patients. Anti-HCV S/Co value >1 was detected in 61 (12.3%) of 495 patients who received HD and were shown not to have HCV and was considered a false positive (Figure 1).

The median age of patients in the HCV-RNA(+) group was found to be higher than that of the HCV-RNA(-) group, and this difference was statistically significant (p<0.018). When examining gender distribution, no statistically significant difference was found between the two groups of patients (p=0.453). Anti-HCV median levels of patients with HCV-RNA(+) results were statistically significantly higher than patients with HCV-RNA(-) results (p<0.0001) (Table 1).

**Figure 2:**
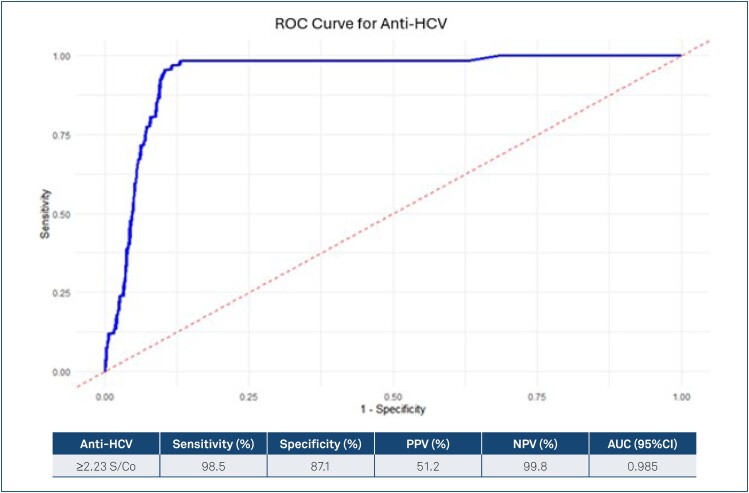
Receiver operating characteristic analysis of anti-hepatitis C virus signal-to-cutoff values according to hepatitis C virus RNA results.

In the ROC curve analysis, HCV-RNA was considered the gold standard to determine the best threshold value, and the most suitable anti-HCV S/Co value was found to be 2.23. The sensitivity was 98.5%, the specificity was 87.1%, and PPV: 51.2% and NPV: 99.8% were calculated (Figure 2).

Genotype-1 was detected in 49 of 57 patients (85.96%), and the most common subtype was genotype 1b (n=43). Genotype-4 (10.52%) was detected in six patients, and genotype-3 (3.52%) in two patients. Genotype 2/5/6/7 or mixed type could not be determined in any patient. There was no statistically significant difference between genotype-1 and other genotypes in terms of age, anti-HCV, and HCV-RNA median values (p=0.8845, p=0.3086, and p=0.1075, respectively).

## DISCUSSION

Hepatitis C virus infection is common among HD patients worldwide^9^. HCV infection affects one in five patients receiving HD treatment and is associated with higher mortality^10^. Regional studies show a relatively high prevalence of HCV among HD patients in countries. These studies show a prevalence of 10.5% in Brazil, 25.3% in the Middle East, 33.5% in northern India, 50% in Egypt, 54% in Syria, less than 5% in northern Europe, less than 5% in most countries in southern Europe, and about 10% in the United States, 28% in Africa, and 48.5% in low-income countries^7,8,10^. In our study, the prevalence of HCV in hemodialysis patients in Turkey was found to be 12.07%. This prevalence rate is slightly higher than the Eastern European data and lower than the Middle Eastern countries. This result was considered to be compatible with the geographical location of Turkey, which is located at the junction of Asia and Europe. Considering that the prevalence of HCV in the general population is around 1% in studies conducted in Turkey in the same years, it can be said that the prevalence in HD patients is higher^2^.

In 2014, the Turkish Society of Nephrology reported that the prevalence of HCV in HD patients in Turkey was 2.7%^11^. In our study, the prevalence of HCV in HD patients in 2021–2024 was 12.07%, which is quite high compared to the Turkish Society of Nephrology data of 2011. It was thought that this result may be related to the migration to our country after 2011, especially from countries with high HCV prevalence, such as Syria.

In our study, true HCV patients were older than non-HCV patients. In addition, the mean age of the patients in our study was higher compared to other studies on the same subject in our country. This may be due to the increase in the rate of chronic kidney disease with aging and the fact that all of our study patients were individuals with chronic kidney disease^6,10,12^.

The Centers for Disease Control and Prevention has allowed laboratories to develop their own HCV diagnostic algorithms to reduce false-positive anti-HCV test results, especially in populations with low HCV prevalence^4^. In studies conducted for this purpose, anti-HCV test results were compared with various verification methods, and threshold values to detect the actual viremia in patients were investigated. The most appropriate anti-HCV S/Co ratio ROC values for differentiating true positive HCV infection were ≥5, ≥7.13, ≥10.86, and ≥15.4, respectively^13-16^. However, it was observed that there was no comprehensive study in the literature on this subject in hemodialysis patients, who are considered to be at high risk for HCV transmission and whose prevalence of the disease is known to be more common than the general population. Our study is the first comprehensive study on this subject. In our study, the anti-HCV S/Co value was ≥2.23 in HD patients in the ROC curve analysis (Figure 2). In our study, lower anti-HCV S/Co values were obtained compared to other studies. It was thought that the other study results reflected the general population, were performed in immunocompetent patients, and decreased antibody response in patients receiving hemodialysis treatment may be associated with a lower S/Co ratio.

According to the results of our study, HCV-RNA was not detected in any of the patients with an anti-HCV S/Co ratio between 1 and 2.23. In patients with anti-HCV values between 1 and 2.23 and positive results, false positivity or cross-reactivity of antibodies caused by other recent viral infections may be considered.

Immunocompromised patients, such as hemodialysis patients, organ transplant recipients, and people with advanced human ımmunodeficiency virus infection, may have higher false-negative rates in antibody tests than immunocompetent patients^17^. However, contrary to this information, we did not find false negative anti-HCV results in 563 HD patients in our study. All of the anti-HCV negative patients had negative HCV RNA test results. Therefore, we emphasize that anti-HCV negative results are reliable in HD patients.

HCV has been classified into 8 genotypes and 105 subtypes^18^. According to a meta-analysis of 407 studies with 1,302,167 participants, HCV genotype 1 was the most common in all regions except Africa. In hemodialysis patients worldwide, the most common HCV genotype was genotype 1b (33.5%), followed by genotypes 1a (22.8%), 3 (8.2%), 2 (6%), 4 (5%), and 6 (2.4%). According to the subgroup analysis by country economic status, HCV genotype 1b predominates in high- and middle-income countries with prevalence rates of 35.4 and 31.6%, respectively. HCV genotype 6 (2.9%) has only been detected in low-income countries^10^.

In the HCV genotyping studies conducted in our country, genotype 1 was found most frequently^15,19^. While the rate of genotype 1 was at the highest level before 2010, it is observed that the rate of genotype 1 decreased in the studies conducted after 2010. In studies conducted in our country in the last decade, it has been emphasized that there has been an increase in genotype 3/4 and mixed genotypes due to the increased inclusion of foreign patients^20^. In our study, genotype 1 was the most common genotype detected in hemodialysis patients, and genotype 4 was the second most common (10.5%). It is thought that genotype 4 was first detected in Turkey in 2011, that it is the dominant genotype in Syria, and that the rate of genotype 4 may have increased in recent years with the influx of refugees to Turkey.

The retrospective nature of our study and the inability to evaluate transmission routes and risk groups are important limitations. In addition, the presence of anti-HCV antibodies in the absence of HCV-RNA may be the result of a past resolved infection or a false-positive antibody detection. As is known, spontaneous clearance may occur in hepatitis C infection (15–25%). In our study, this rate could not be calculated precisely because sufficient information about the patient's history was not available. This situation can be considered as another limitation of our study.

## CONCLUSION

HCV prevalence was found to be higher in patients receiving hemodialysis treatment compared to the general population. Anti-HCV negative results are highly reliable in HD patients. If anti-HCV S/Co ≥2.23, confirmation with direct HCV RNA testing is recommended. If the anti-HCV S/Co value is detected between 1 and 2.23, false positivity should be considered as a priority. In cases of high clinical suspicion for HCV, repeat anti-HCV testing with a new sample is recommended, and confirmation with HCV RNA is recommended if seropositivity is detected for the second time.

## Data Availability

The datasets generated and/or analyzed during the current study are available from the corresponding author upon reasonable request.
